# Outcomes of Acute Kidney Injury in Melioidosis: A Systematic Review and Meta-Analysis

**DOI:** 10.3390/life15071108

**Published:** 2025-07-15

**Authors:** Wiyada Kwanhian Klangbud, Moragot Chatatikun, Sa-ngob Laklaeng, Jitabanjong Tangpong, Pakpoom Wongyikul, Phichayut Phinyo, Jongkonnee Thanasai, Supphachoke Khemla, Chaimongkhon Chanthot, Atthaphong Phongphithakchai

**Affiliations:** 1 Medical Technology Program, Faculty of Science, Nakhon Phanom University, Nakhon Phanom 48000, Thailand; wiyadakwanhian@gmail.com; 2 School of Allied Health Sciences, Walailak University, Nakhon Si Thammarat 80160, Thailand; moragot.ch@wu.ac.th (M.C.); sumoun2528@gmail.com (S.-n.L.); rjitbanj@wu.ac.th (J.T.); 3 Research Excellence Center for Innovation and Health Products (RECIHP), Walailak University, Nakhon Si Thammarat 80160, Thailand; 4 Center for Clinical Epidemiology and Clinical Statistics, Faculty of Medicine, Chiang Mai University, Chiang Mai 50200, Thailand; aumkidify@gmail.com (P.W.); phichayutphinyo@gmail.com (P.P.); 5 Department of Biomedical Informatics and Clinical Epidemiology (BioCE), Faculty of Medicine, Chiang Mai University, Chiang Mai 50200, Thailand; 6 Faculty of Medicine, Mahasarakham University, Mahasarakham 44000, Thailand; jongkonnee@msu.ac.th; 7 Division of Infectious Diseases, Department of Internal Medicine, Nakhon Phanom Hospital, Nakhon Phanom 48000, Thailand; sup.mednkp@gmail.com; 8 Project for the Establishment of the Faculty of Medicine, Nakhon Phanom University, Nakhon Phanom 48000, Thailand; chaimongkhon251269@gmail.com; 9 Nephrology Unit, Division of Internal Medicine, Faculty of Medicine, Prince of Songkla University, Songkhla 90110, Thailand

**Keywords:** melioidosis, *Burkholderia pseudomallei*, acute kidney injury, AKI, mortality, clinical outcome

## Abstract

**Background:** Melioidosis is a severe infectious disease caused by *Burkholderia pseudomallei*, with high mortality rates, particularly in severe cases complicated by acute kidney injury (AKI). **Objective:** The objective of this study was to systematically review and quantitatively synthesize the impact of AKI on mortality and other clinical outcomes—including ICU admission and the need for renal replacement therapy (RRT)—in patients with melioidosis. **Methods:** A systematic search was conducted in PubMed, Scopus, and Embase up to 16 May 2025. Studies reporting mortality, ICU admission, or RRT use in patients with AKI were included. A random-effects meta-analysis was performed to estimate the odds ratio (OR) for mortality associated with AKI. **Results:** Twenty-nine studies (380 patients) were included. AKI occurred in 123 patients (32.4%). The pooled analysis revealed that AKI patients had a significantly higher mortality risk than non-AKI patients (OR = 23.37; 95% CI: 13.97–39.10; *p* = 0.0082), with no significant heterogeneity (I^2^ = 0%). Sensitivity analysis confirmed the robustness of this association. ICU admission and RRT data were frequently reported but were not suitable for meta-analysis due to insufficient data. **Conclusions:** AKI is a serious complication in melioidosis, significantly increasing the risk of mortality. Early recognition and aggressive management of AKI in melioidosis may be critical to improving clinical outcomes.

## 1. Introduction

*Burkholderia pseudomallei* is a motile, Gram-negative, facultative intracellular bacillus and the causative agent of melioidosis, a disease of increasing global importance. It is an environmental saprophyte that thrives in soil and stagnant water, particularly in tropical and subtropical regions. Human infection occurs primarily via percutaneous inoculation, inhalation, or ingestion, especially during the rainy season or extreme weather events such as typhoons or floods [1]. Melioidosis is endemic in Southeast Asia and Northern Australia but has also been reported in South Asia, the Middle East, Africa, and more recently in the Americas [2]. Clinical manifestations are highly variable, ranging from asymptomatic infection and localized skin ulcers to fulminant septicemia with multi-organ failure. Diabetes mellitus is the most common risk factor, followed by chronic kidney disease, hazardous alcohol use, and immunosuppression [3].

One of the severe and underrecognized complications of melioidosis is acute kidney injury (AKI). AKI can result from direct renal invasion by *B. pseudomallei*, systemic inflammation, septic shock, or nephrotoxic treatments (such as aminoglycosides) used during management [4]. Clinical manifestations of AKI in melioidosis range from transient elevations in serum creatinine to oliguric or anuric renal failure, and in some cases, the need for renal replacement therapy (RRT). Patients with AKI often have concurrent complications, such as pneumonia, hepatic dysfunction, or hematologic abnormalities, which exacerbate disease severity. The requirement for renal replacement therapy (RRT) has been reported in several cases, highlighting the burden of critical illness in this patient population [5]. Despite its clinical importance, the true incidence and impact of AKI in melioidosis remain unclear. Studies have used a range of diagnostic criteria, including the Acute Kidney Injury Network (AKIN) and the Kidney Disease: Improving Global Outcomes (KDIGO) definitions, leading to considerable heterogeneity in reported outcomes [6]. In the broader context of sepsis, AKI is a well-established predictor of poor prognosis. Patients with sepsis-associated AKI experience higher mortality, longer ICU and hospital stays, and a greater likelihood of long-term renal dysfunction [7]. Similarly, limited data from melioidosis studies suggest a strong association between AKI and increased mortality. For example, Chou et al. (2007) reported that 47.4% of melioidosis patients with AKI died, while none without AKI experienced mortality [4]. Prabhu et al. (2021) also found that AKI was independently associated with increased risk of death and ICU admission [5]. However, to date, no systematic review or meta-analysis has quantified the association between AKI and clinical outcomes in melioidosis.

We hypothesized that AKI is associated with increased risk of mortality and adverse clinical outcomes in patients with melioidosis. Therefore, we conducted a systematic review and meta-analysis to assess whether the presence of acute kidney injury (AKI) in patients with melioidosis is associated with increased mortality. Understanding the prognostic implications of AKI in melioidosis may support early identification, risk stratification, and targeted interventions to reduce morbidity and mortality in this high-risk group.

## 2. Materials and Methods

### 2.1. Protocol Registration

This systematic review and meta-analysis were conducted according to the Preferred Reporting Items for Systematic Reviews and Meta-Analyses (PRISMA) guidelines [8]. The review protocol was prospectively registered with the International Prospective Register of Systematic Reviews (PROSPERO; registration number: CRD420251052619).

### 2.2. Search Strategy

We systematically searched three major electronic databases—PubMed, Scopus, and Embase—from their inception to 16 May 2025. The search terms included combinations of Medical Subject Headings (MeSH) and keywords related to *Burkholderia pseudomallei*, melioidosis, acute kidney injury, and clinical outcomes. The complete search strategies for each database are provided in Supplementary Table S1.

To ensure comprehensive coverage, we also examined the reference lists of all included studies and relevant reviews. Additionally, we searched gray literature sources, such as Google Scholar and reference lists from papers, to identify any potentially eligible reports not indexed in the traditional databases. Only studies published in English were included in this review.

### 2.3. Inclusion and Exclusion Criteria

We included studies that met the following criteria. Population: Human subjects diagnosed with culture-confirmed or clinically diagnosed melioidosis. Exposure: Presence of acute kidney injury, as defined by the authors of the included studies. Comparator: Patients with melioidosis without AKI. Outcomes: At least one of the following reported outcomes—mortality (in-hospital or 30-day), ICU admission, or requirement for renal replacement therapy (RRT). Study design: Case reports, case series, retrospective cohort studies, or prospective studies that provided extractable individual or grouped outcome data.

We excluded review articles, animal studies, editorials, conference abstracts without sufficient data, and non-English publications. Case reports were excluded only if they lacked outcome data relevant to AKI.

### 2.4. Study Selection

All identified records were imported into a reference management software program (EndNote), and duplicates were removed. Two independent reviewers (W.K.K. and A.P.) screened titles and abstracts against the eligibility criteria. Full texts of potentially relevant studies were then retrieved and assessed for inclusion. Discrepancies were resolved by discussion or by consultation with a third reviewer.

### 2.5. Data Extraction

Data were extracted independently by two reviewers, using a standardized data extraction form. Extracted variables included first author, year of publication, country, sample size, number of AKI cases, definition of AKI, mortality in AKI and non-AKI groups, ICU admission rates, RRT use, and relevant clinical characteristics (e.g., comorbidities and organ dysfunction). Definitions of AKI varied across studies, with some using standardized criteria (e.g., AKIN or KDIGO) and others relying on clinical judgment or unspecified parameters. Where data were unclear or missing, authors were contacted when possible. Any disagreements were resolved by consensus.

### 2.6. Quality Assessment

We assessed study quality using the Joanna Briggs Institute (JBI) critical appraisal tools, selecting checklists based on study design. The appropriate JBI checklists were applied to case reports (*n* = 19; 8 items), case series (*n* = 8; 10 items), and cohort studies (*n* = 2; 11 items), evaluating factors such as patient selection, diagnostic clarity, outcome reporting, and risk of bias.

Two reviewers (W.K.K. and A.P.) independently rated each item as “Yes,” “No,” “Unclear,” or “Not applicable,” with disagreements resolved by discussion. Studies were not excluded based on quality, but ratings were used to support the interpretation of results. Based on the number of criteria met, studies were classified as having low, moderate, or high risk of bias.

### 2.7. Statistical Analysis

Pooled odds ratios (ORs) were calculated using a random-effects model. Log-transformed ORs and their standard errors were used to compute 95% confidence intervals (CIs). The between-study variance (*Tau*^2^) was estimated to account for variability across studies. The *I*^2^ statistic was used to assess the degree of heterogeneity between studies. Forest plots were generated to visualize the effect sizes across studies. All analyses were performed using R software with the “meta” package (R Foundation for Statistical Computing, Vienna, Austria).

For outcomes with insufficient data (e.g., ICU admission and RRT use), a narrative synthesis was conducted. A sensitivity analysis was performed by excluding individual studies to assess the robustness of the primary outcome. The results of these sensitivity tests, which confirmed the consistency of the primary findings, are provided in Supplementary Figures S1 and S2.

### 2.8. Bayesian Re-Analysis of Risk Difference (RD)

To address concerns regarding studies with zero events in both treatment and control arms, we re-analyzed the data using a Bayesian Generalized Linear Model (GLM) with the *stan_glm* function from the *rstanarm* package in R. A Gaussian family with an identity link was used to model the Risk Difference (RD) across studies, specifying the formula rd~1 and generating 4000 posterior samples via Markov Chain Monte Carlo (MCMC) sampling. This approach effectively handled zero-event studies, offering more reliable RD estimates [9,10].

## 3. Results

### 3.1. Included Studies

**Figure 1:**
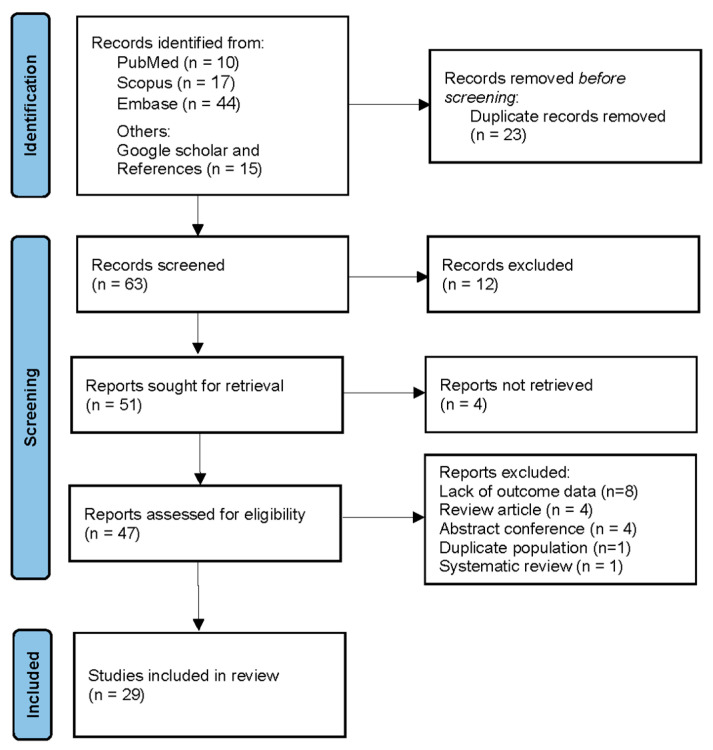
PRISMA flow diagram.

A total of 86 records were identified through database searches: 10 from PubMed, 17 from Scopus, and 44 from Embase. An additional 15 records were identified through Google Scholar and references. After removing 23 duplicate records, 63 records remained for screening. Of these, 12 were excluded based on title and abstract review. Full-text reports were sought for 51 records, but 4 reports could not be retrieved. After assessing the eligibility of 47 reports, 8 were excluded due to lack of outcome data, 4 were review articles, 4 were conference abstracts, 1 had a duplicate population, and 1 was a systematic review. Ultimately, 29 studies were included in the systematic review and meta-analysis. The PRISMA flow diagram is presented in Figure 1. Additionally, as shown in Supplementary Table S2, 18 studies were excluded from the final analysis for reasons such as duplicate populations, lack of outcome data, review articles, and conference abstracts.

### 3.2. Characteristics of Included Studies

A total of 29 studies were included in this systematic review and meta-analysis, comprising 380 patients with melioidosis, of whom 123 (32.4%) developed AKI. The studies were conducted across a wide geographical distribution, including Southeast Asia (e.g., Thailand, Malaysia, Singapore, and Vietnam), South Asia (India and Sri Lanka), the Middle East (Saudi Arabia and Oman), and Western countries (United States, Australia, and the Netherlands).

Study designs included case reports (*n* = 19), case series (*n* = 8), and retrospective cohort studies (*n* = 2). The sample sizes varied from single-patient case reports to a large retrospective study with 164 patients. The reported incidence of AKI ranged widely from 3.7% to 100%, with AKI being more common in studies involving critically ill patients.

Mortality among patients with AKI varied by study and ranged from 0% to 100%, with several reports noting markedly higher mortality in the AKI group compared to those without AKI. The need for RRT was reported in several studies, reflecting the severity of renal dysfunction. Organ dysfunction beyond the kidneys was common, including pulmonary, hepatic, and neurological involvement.

**Table 1:** Characteristics of included studies.

Author (Year) [Ref.]	Country	Sample Size (*n* = 380)	AKI Cases (*n* = 123)	AKI %	Mortality (AKI)	Mortality (no AKI)	ICU Admission	RRT Required	Organ Dysfunction	Risk Factor	AKI Definition	Notes
Alhatmi (2020) [11]	Saudi Arabia	2	1	50	100% (Case 1)	0% (Case 2)	1	1	Multi-organ failure	Travel to Thailand (Case 1), India (Case 2)	Not explicitly defined, clinical evidence	Case 1: Fulminant sepsis, ECMO support; Case 2: Treated successfully with antibiotics.
Amali (2024) [12]	Singapore	1	1	100	0% (Survived)	Not applicable	Yes (ECMO support)	Yes	Multi-organ dysfunction, including lungs, spleen, liver (hepatosplenic abscesses)	CASP4 mutation (R344W), no diabetes or other comorbidities	Acute presentation with renal dysfunction requiring ECMO support	Persistent *B. pseudomallei* infection, treated with recombinant IFN-γ leading to successful outcome and discharge.
Arya (2021) [13]	United States	1	1	100	0% (Survived)	Not applicable	Yes	Not specified	Multiple organ involvement, including lungs, spleen, kidneys (severe sepsis)	Type-2 diabetes, hyperlipidemia, non-alcoholic fatty liver disease, prior SARS-CoV-2 infection, recent travel to Bangladesh	Clinical evidence of acute renal dysfunction	Complex case with delayed diagnosis, multiple organ abscesses, treated with meropenem and TMP-SMX, eventual recovery.
Boyle (2024) [14]	Australia	8	2	25	0/2 (0%)	0/5 (0%)	6/8 (75%)	Not specified	Multiple organ systems, including aortic, renal, pulmonary, splenic involvement	Vascular disease, diabetes, chronic kidney disease, chronic lung disease, alcohol use	Not explicitly defined, clinical diagnosis of acute renal dysfunction	Complex cases of mycotic aneurysm due to *Burkholderia pseudomallei*, delayed diagnosis common, management includes surgical intervention and long-term antibiotics, high morbidity and mortality noted.
Chang (2020) [15]	Malaysia	1	1	100	0% (Survived)	Not applicable	Not specified	No	Splenic abscess, renal dysfunction, sepsis	Pregnancy, no other comorbidities	Mild renal impairment with raised serum creatinine at 129 µmol/L	Case of a young pregnant woman with bacteraemic melioidosis and splenic abscesses, eventual spontaneous abortion.
Chanvitan (2019) [16]	Thailand	27	1	3.7	100% (1/1)	9/26 (34.6%)	Not specified	Not specified	Sepsis, pneumonia, soft tissue infection, splenic and hepatic abscesses	Diabetes, thalassemia, renal disease in a minority of cases	Increase in serum creatinine ≥ 0.3 mg/dL or 1.5-fold from baseline	Pediatric cohort, significant hepatic and splenic abscesses as diagnostic clues, AKI rare but fatal in one case.
Che Rahim (2019) [17]	Malaysia	1	1	100	0% (Survived)	Not applicable	Yes	Yes (dialysis)	Septic shock, respiratory failure, hepatic dysfunction, renal dysfunction	Systemic lupus erythematosus, immunosuppression	Acute renal failure requiring dialysis	Young female patient with SLE, developed severe sepsis and multi-organ failure, successfully recovered with intensive treatment including immunosuppression and antibiotics.
Chou (2007) [4]	Taiwan	30	19	63.3	9/19 (47.4%)	0% (0/11)	14/30 (46.7%)	Not specified	Sepsis, pneumonia, respiratory failure, renal failure	Diabetes mellitus, chronic renal disease, excessive alcohol consumption, malignancy, cardiovascular disease	Reduction in estimated creatinine clearance of 50% or need for RRT	Study of bacteremic melioidosis in Taiwan post-typhoon outbreak; high mortality in patients with AKI, often associated with pneumonia and septic shock.
Cossaboom (2020) [18]	United States	1	1	100	0% (Survived)	Not applicable	Yes	Yes (CRRT)	Respiratory failure, sepsis	Type 2 diabetes, unilateral renal agenesis, rural water exposure	Clinical evidence with renal failure	Melioidosis acquired from environmental exposure in Texas; patient recovered with treatment.
Fairhead (2020) [19]	Australia	1	1	100	0% (Survived)	Not applicable	Yes	Not specified	Pneumonia, discitis, osteomyelitis	Rheumatoid arthritis, chronic lung disease, immunosuppression with etanercept	Elevated creatinine on admission	Polymicrobial bacteremia (melioidosis and Acinetobacter), improved with antibiotics.
Ganesan (2019) [20]	India	7	6	85.7	50% (3/6)	0% (0/1)	Not specified	Not specified	Renal dysfunction, hepatic dysfunction, sepsis, metabolic derangements	Diabetes, alcoholism	Elevated renal parameters and clinical evidence	Case series of melioidosis with high mortality among AKI cases; slow microbiological clearance and persisting radiological abnormalities were noted.
Gouse (2017) [21]	India	24	2	8.3	50% (1/2)	0% (0/22)	Not specified	Not specified	Musculoskeletal involvement, septic arthritis, osteomyelitis, intramuscular abscesses	Diabetes, thalassemia, sickle cell anemia, chronic renal disease	Clinical evidence with acute renal failure	Largest musculoskeletal melioidosis series from India, surgical management resulted in good outcomes.
Gulati (2022) [22]	United States (Vietnam origin)	1	1	100	100% (Died)	Not applicable	Yes	No (died before)	Sepsis, multi-organ dysfunction, ARDS	Diabetes, HIV infection, history of travel to Vietnam	Acute renal failure with sepsis	First reported case of latent melioidosis activation by COVID-19; rapid deterioration despite treatment.
Gunasena (2023) [23]	Sri Lanka	1	1	100	0% (Survived)	Not applicable	Yes	Yes (intermittent hemodialysis)	Pulmonary hemorrhage, sepsis, jaundice	Farmer, environmental exposure	Acute renal dysfunction, oliguric AKI	Co-infection with leptospirosis and melioidosis, recovered with antibiotics, dialysis, and plasma exchange.
Gupta (2021) [24]	India	11	3	27.3	33.3% (1/3)	0% (0/8)	Not specified	Not specified	Osteoarticular melioidosis with systemic involvement	Diabetes, trauma, immunosuppression	Clinical evidence of acute renal dysfunction	Combination of osteomyelitis and arthritis, some patients had pulmonary involvement, treated with meropenem/ceftazidime and cotrimoxazole.
Hin (2012) [25]	Malaysia	4	1	25	100% (1/1)	66.7% (2/3)	Yes	Yes (hemodialysis)	Sepsis, multi-organ failure, pneumonia, hepatic dysfunction	Diabetes, leptospirosis co-infection, rescue operation exposure	Acute renal failure with sepsis	Cluster of cases among rescuers exposed to contaminated water, co-infection with leptospirosis confirmed by PCR.
Jagtap (2017) [26]	India	9	1	11.1	100% (1/1)	14.3% (1/7)	Not specified	Not specified	Liver abscess, splenic abscess, pancreatic abscess, empyema, SBP	Diabetes, alcoholism	Acute-on-chronic liver failure with renal dysfunction	GI manifestations of melioidosis, unusual presentation including pancreatitis and SBP, high mortality with severe liver disease.
Jang (2015) [27]	Korea	1	1	100	0% (Survived)	Not applicable	Yes	Yes (due to worsening renal function)	Mycotic aneurysm with multi-organ embolism	Travel to Thailand, IgA nephropathy, gout, hypertension	Acute renal dysfunction during hospital course	Identified *B. pseudomallei* by 16S rRNA sequencing, surgical intervention with aortic repair, good recovery.
Lim (2022) [28]	Malaysia	1	1	100	100% (Died)	Not applicable	Yes	Yes (hemodialysis)	Multi-organ failure, sepsis, ARDS, hepatic dysfunction	Uncontrolled diabetes, co-infection with leptospirosis, panhypopituitarism	Acute renal failure with sepsis and shock	Late diagnosis due to non-specific presentation, death from multi-organ failure despite aggressive treatment.
Liu (2014) [29]	Singapore	74	9	12.2	15.9% (Approx.)	3.3% (Approx.)	Not specified	Not specified	Sepsis, hypotension, respiratory distress, renal impairment	Type II diabetes, sulfonylurea treatment	Renal impairment with need for RRT	Sulfonylurea usage linked to severe septic complications and immune suppression.
Loh (2017) [30]	Australia	1	1	100	0% (Survived)	Not applicable	Yes	Yes (dialysis)	Sepsis, respiratory failure, cerebral abscess, temporal lobe involvement	Pig hunting, exposure to soil and environmental pathogens	Acute renal failure with raised creatinine (241 µmol/L)	Complex case with delayed diagnosis, eventually recovered with prolonged antibiotics.
Meraj (2019) [31]	United States (Filipino origin)	1	1	100	0% (Survived)	Not applicable	Yes	Yes (meropenem failure)	Persistent bacteremia sepsis	Travel to endemic areas, ceftazidime-resistant strain	Persistent bacteremia with renal involvement	Prolonged meropenem treatment required, eventual recovery.
Morelli (2015) [32]	Netherlands (Gambia travel)	1	1	100	0% (Survived)	Not applicable	Yes	Yes (eventual hemodialysis)	Sepsis, prostatic abscess, ESRD	Travel to Gambia, environmental exposure	Acute renal failure progressing to ESRD	Prostatic abscess, recovery with ceftazidime, but renal failure persisted.
Prabhu (2021) [5]	India	164	59	35.98	32.2%	5.7%	37.3% (AKI), 13.3% (non-AKI)	8/59 (13.6%)	Sepsis, bacteremia, shock, multi-organ dysfunction	CKD, bacteremia, shock	AKIN criteria	AKI associated with higher mortality and ICU care; survivors showed kidney recovery.
Stewart (2021) [33]	United States (Arizona)	1	1	100	0% (Survived)	Not applicable	Yes	Not specified	Sepsis, pneumonia, multiple abscesses	Unknown environmental exposure	Not directly defined, clinical evidence	First autochthonous case in the U.S., delayed diagnosis, recovery with antibiotics.
Tamtami (2017) [34]	Oman	1	1	100	100% (Died)	Not applicable	Yes	Yes	Severe sepsis, ARDS, multi-organ failure	Occupational exposure in Laos/Cambodia, diabetes mellitus	Acute renal failure with multi-organ dysfunction	Imported case of melioidosis with *B. pseudomallei* isolated from blood; rapid deterioration despite intensive therapy.
Tran (2022) [35]	Vietnam	3	2	66.7	100% (2/2)	100% (1/1)	Yes (for all)	Not specified	Liver dysfunction, renal dysfunction, sepsis	Contaminated borehole water (*B. pseudomallei* ST541)	Elevated creatinine, clinical evidence	Cluster of 3 children from 1 family; *B. pseudomallei* traced to household borehole water; severe outcomes with liver and kidney involvement.
Wadwekar (2018) [36]	India	1	1	100	0% (Survived)	Not applicable	Yes	Not specified	Sepsis, multiple abscesses, splenic rupture	Diabetes mellitus, soil exposure	Acute renal dysfunction with sepsis	Case report of melioidosis presenting with sepsis and splenic rupture, survived after aggressive treatment.
Warapitiya (2021) [37]	Sri Lanka	1	1	100	100% (Died)	Not applicable	Yes	Yes (dialysis)	Severe sepsis; multi-organ failure, including renal and hepatic failure	Cut injury in paddy field, environmental exposure	Acute renal failure requiring dialysis	Severe sepsis with melioidosis, leading to multi-organ failure and death despite ICU support and broad-spectrum antibiotics.

AKI, acute kidney injury; RRT, renal replacement therapy; CKD, chronic kidney disease; ST, sequence type; ICU, intensive care unit.

Risk factors associated with AKI in melioidosis included diabetes mellitus, chronic kidney disease, sepsis, and environmental exposures such as travel to endemic areas or contaminated water sources. AKI definitions varied, with some studies applying standard clinical criteria, such as serum creatinine elevation [15,16] or AKIN/KDIGO guidelines [23,25], while others relied on clinical judgment or did not provide explicit definitions.

Overall, the included studies demonstrate the heterogeneity of AKI presentation in melioidosis and its significant association with increased morbidity and mortality, particularly in cases requiring ICU care or RRT (see Table 1 for detailed study characteristics).

### 3.3. Pooled Odd and Heterogeneity

**Figure 2:**
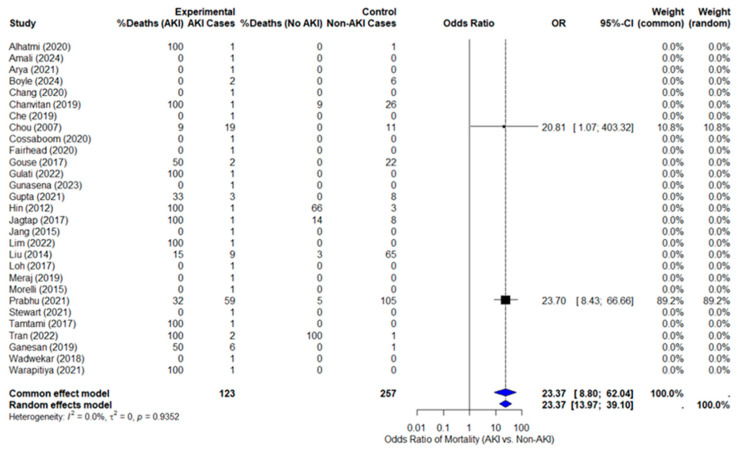
The forest plot.

Data extraction was completed for mortality outcomes, but ICU admission and RRT data were insufficient for pooled analysis. The pooled odds of death in melioidosis patients with AKI are 23 times higher than in patients without AKI (OR = 23.37, 95% CI: 13.97–39.10, *p* = 0.0082), as shown in Figure 2 with the forest plot. This result is statistically significant, and the confidence interval does not cross 1, confirming the strength of the association. *I*^2^ for mortality was 0%, indicating low heterogeneity across included studies. In addition, *Tau*^2^ = 0 also indicates low between-study variance. However, only two studies contributed usable data: Prabhu (2021) [5] dominated 89% of the weight, while Chou (2007) [4] contributed ~11%, with a very wide CI.

To further address concerns regarding studies with zero events in both treatment and control arms, a Bayesian re-analysis was conducted. The results are presented in Supplementary Figure S3. The estimates indicate that the posterior mean for the intercept is 0.5, with no variability (SD = 0.0), suggesting that the RD is consistent across the two studies. The sigma value, representing the variability in RD, is 0.0, further supporting the absence of variability between the studies. The mean posterior predictive distribution (mean_PPD) is also 0.5, reflecting the predicted RD from the posterior distribution. The MCMC diagnostics show convergence with Rhat values of 1.0, and the effective sample sizes for the parameters are satisfactory, ranging from 677 for the intercept to 1173 for the mean_PPD. These findings suggest that the Risk Difference (RD) is consistent across the studies and can be used for decision-making in research. However, the absence of variability (sigma = 0.0) indicates that the data from the two studies by Chou (2007) [4] and Prabhu (2021) [5] are highly consistent.

### 3.4. Sensitivity Analysis

To assess the influence of individual studies on the overall estimate, a sensitivity analysis was performed by sequentially excluding individual studies to test the robustness of the primary outcome. Initially, we excluded the study by Prabhu et al. (2021) [5], which contributed 89.2% of the weight in the primary meta-analysis. When only the study by Chou et al. (2007) [4] was included, the odds ratio remained elevated (OR = 20.81; 95% CI: 1.07–403.32). The forest plot for this analysis is shown in Supplementary Figure S1. Further sensitivity analyses were conducted by excluding the study by Chou et al. (2007) [4]. The odds ratio (OR = 23.0; 95% CI: 8.43–66.66) remained similar to the pooled OR, as shown in Supplementary Figure S2. While the estimate remained consistent, the wide confidence interval and reliance on a single study limit the interpretability of this result. Together, these analyses suggest that the primary outcome remains relatively robust, but the uncertainty in the estimates, particularly when based on a small number of studies, warrants caution in interpreting the findings.

### 3.5. Qualities of Included Studies

The methodological quality of all 29 included studies was evaluated using JBI critical appraisal tools appropriate to each study design: case reports (*n* = 19), case series (*n* = 8), and cohort studies (*n* = 2). Most studies were rated as having a low-to-moderate risk of bias. Among case reports, 68% (*n* = 13) scored ≥ 7/8, with clear reporting of clinical presentation, diagnosis, and outcomes. Some lacked detail on adverse events or follow-up. Of the case series, 63% (*n* = 5) met at least 80% of quality criteria, though several had unclear inclusion methods or incomplete reporting of baseline data. Both cohort studies were of relatively high quality. Prabhu (2021) [5] scored 10/11, with well-defined AKI criteria and adjustment for confounders, while Chou (2007) [4] scored 9/11 but lacked detailed control of confounding. Full quality ratings and comments are presented in Supplementary Table S3.

## 4. Discussion

This systematic review and meta-analysis provide a comprehensive synthesis of current evidence regarding the impact of AKI on outcomes in patients with melioidosis. Across 29 studies involving a total of 380 patients, AKI was identified in approximately one-third of cases (32.4%). Importantly, the presence of AKI was associated with a significantly increased risk of mortality, with a pooled odds ratio of 23.37 (95% CI: 13.97–39.10). This finding emphasizes the severity of AKI as a complication of melioidosis and highlights its prognostic importance.

Our findings align with the broader literature on sepsis-associated AKI, which consistently demonstrates that AKI independently predicts worse outcomes, including higher mortality, prolonged ICU stays, and greater dependence on organ support [7]. In melioidosis, AKI may result from several pathophysiological mechanisms, such as direct bacterial invasion, septic shock, systemic inflammation, nephrotoxic medications, and pre-existing renal conditions. These mechanisms resemble those observed in other tropical infectious diseases, like leptospirosis and dengue, where AKI is caused by a combination of hemodynamic instability, tubular toxicity, and immune-mediated injury [5,38,39,40,41]. Some studies in our analysis reported cases requiring renal replacement therapy, particularly in patients with multi-organ failure, underscoring the critical nature of AKI in this population.

Despite consistent evidence of increased mortality, there was substantial heterogeneity in study design, AKI definitions, and outcome reporting. Definitions of AKI varied considerably—only a minority of studies applied standardized criteria like AKIN [5], while others relied on clinical judgment or did not specify criteria at all. This variability likely introduced misclassification bias and limited the comparability of findings across studies. Additionally, data on ICU admission and RRT use were inconsistently reported, precluding pooled analysis of these secondary outcomes. Nonetheless, the low statistical heterogeneity observed in our mortality analysis (*I*^2^ = 0%) suggests a robust association across diverse study settings. Subgroup sensitivity analyses by region, AKI severity, or comorbidities were not possible due to insufficient or inconsistent data reporting. Future studies should focus on standardizing reporting practices to enable more detailed and accurate analyses of AKI in melioidosis.

This study has several limitations. Firstly, the majority of included studies were case reports or small observational studies, which are prone to bias and lack generalizability. Secondly, AKI definitions varied significantly across studies, with few adhering to standardized criteria such as AKIN or KDIGO, potentially leading to misclassification. Thirdly, most studies lacked data on long-term renal outcomes, recovery from AKI, and the timing of renal replacement therapy initiation. Additionally, only two studies provided suitable data for meta-analysis, with one (Prabhu et al., 2021) contributing approximately 89% of the statistical weight [5]. This heavy reliance on a single study limits generalizability and may introduce bias, suggesting that the findings should be interpreted with caution. Fourth, the exclusion of non-English articles may have led to language bias, omitting potentially relevant regional data. We acknowledge this limitation and suggest that future reviews consider multilingual inclusion or translation support to minimize bias. Finally, long-term mortality data (e.g., 60-day or 90-day mortality) were not available, so our analysis focused on in-hospital and 30-day mortality, potentially underestimating the true mortality burden associated with AKI.

While the overall quality of the included studies was assessed as low to moderate using JBI criteria, many studies lacked sufficient detail on adverse events or follow-up, limiting the ability to draw strong conclusions. Inconsistent reporting of clinical risk factors, such as diabetes, chronic kidney disease, and sepsis, further complicates the interpretation of results. The absence of multivariable analysis prevented the adjustment for potential confounders, suggesting that the observed association between AKI and mortality should be interpreted with caution due to unmeasured or uncontrolled biases.

Our findings align with established research on sepsis-associated AKI, which identifies renal impairment as a key predictor of mortality. The current KDIGO guidelines emphasize early identification, fluid resuscitation, and renal function monitoring in critically ill patients [6,42], which may be particularly relevant in melioidosis-endemic regions. For clinicians, early detection of AKI in melioidosis patients, especially those with sepsis or multi-organ dysfunction, is crucial. Routine renal function monitoring; cautious use of nephrotoxic agents; and timely initiation of supportive therapies, including renal replacement therapy, when necessary, should be prioritized. Given the high mortality risk linked to AKI, patients should be considered for early ICU referral and multidisciplinary care.

This review also highlights critical gaps in the current literature. Prospective studies using standardized definitions and outcome measures for AKI in melioidosis are urgently needed. There is also a limited understanding of long-term renal outcomes and recovery in survivors of melioidosis-associated AKI. Additionally, variations in healthcare infrastructure between endemic and non-endemic regions may significantly influence management, and outcomes were not adequately addressed in the studies reviewed.

## 5. Conclusions

Acute kidney injury is a prevalent and severe complication in melioidosis, significantly increasing mortality risk. Our findings highlight the critical need for early detection, prompt intervention, and vigilant monitoring of renal function in melioidosis patients, especially those with sepsis or pre-existing risk factors for renal impairment. Future research should focus on standardizing AKI definitions in melioidosis, enhancing the quality of prospective data, and investigating interventions to reduce kidney injury and improve survival in this high-risk group.

## Data Availability

Not applicable.

## Supplementary Materials

The following supporting information can be downloaded at https://www.mdpi.com/article/10.3390/life15071108/s1, Figure S1: The forest plot that excludes Prabhu (2021) [5] study; Figure S2: The forest plot that excludes Chou (2007) [4] study; Figure S3: A Bayesian model analysis of Risk Difference (RD) calculated using data from two studies (Chou 2007 [4] and Prabhu 2021 [5]); Table S1: Search strategies; Table S2: Eighteen studies were excluded from the final analysis; Supplementary Table S3: Quality assessment of included studies, using JBI checklists. References [43,44,45,46,47,48,49,50,51,52,53,54,55,56,57,58,59,60] are cited in the supplementary materials.

## Abbreviations

The following abbreviations are used in this manuscript:

AKI	acute kidney injury
AKIN	Acute Kidney Injury Network
ARDS	Acute Respiratory Distress Syndrome
CKD	chronic kidney disease
ECMO	Extracorporeal Membrane Oxygenation
ICU	intensive care unit
KDIGO	Kidney Disease: Improving Global Outcomes
MeSH	Medical Subject Heading
OR	odds ratio
PRISMA	Preferred Reporting Items for Systematic Reviews and Meta-Analyses
SLE	Systemic Lupus Erythematosus
