# Supplementary material for: Outcomes of Acute Kidney Injury in Melioidosis: A Systematic Review and Meta-Analysis
Source: Life (Basel). 2025 Jul 15;15(7):1108. doi: 10.3390/life15071108 (PMC12299289; doi:10.3390/life15071108)
Supplement: Supplementary file 1 [file life-15-01108-s001.zip › Figure S3.pdf]

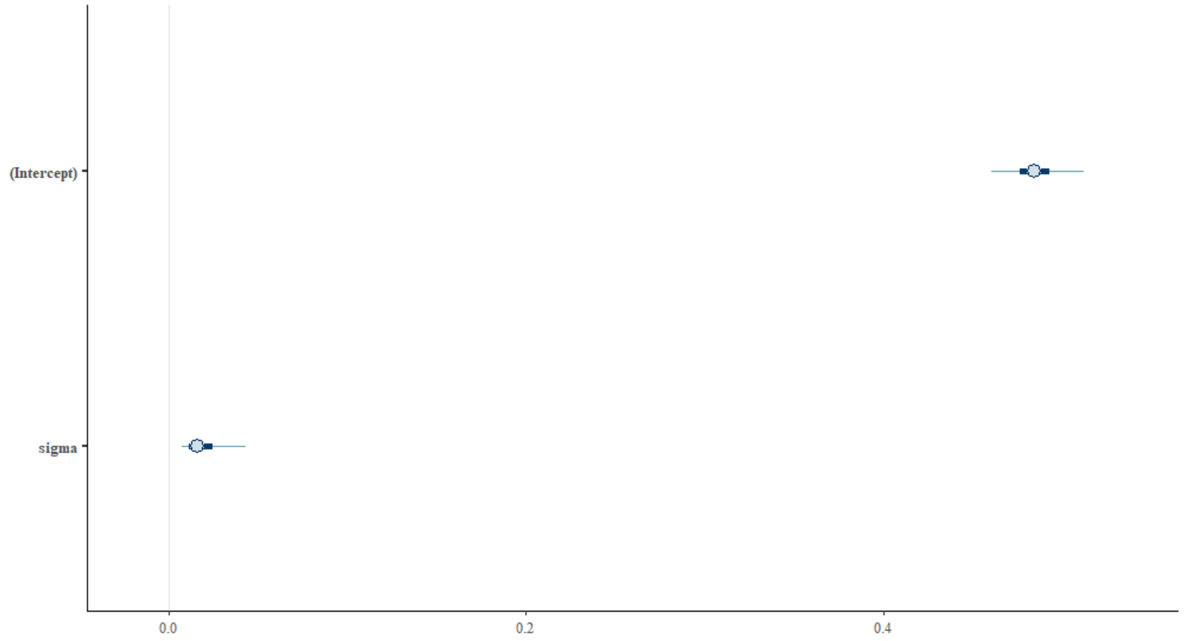

Supplementary Figure S3. A Bayesian model analysis of Risk Difference (RD) calculated using data from two studies (Chou 2007 and Prabhu 2021). The model was implemented using the *stan_glm* function with a Gaussian family and an identity link. The analysis was based on 4000 posterior samples, and the formula used was *rd* ~ 1, meaning the model evaluated the RD with an intercept only. The estimates indicate that the posterior mean for the intercept is 0.5, with no variability (SD = 0.0), suggesting that the RD is consistent across the two studies. The sigma value, representing the variability in RD, is 0.0, further supporting the absence of variability between the studies. The mean posterior predictive distribution (mean_PPD) is also 0.5, reflecting the predicted RD from the posterior distribution. The MCMC diagnostics show convergence with Rhat values of 1.0, and the effective sample sizes for the parameters are satisfactory, ranging from 677 for the intercept to 1173 for the mean_PPD. These findings suggest that the Risk Difference is consistent across the studies and can be used for decision-making in research. However, the absence of variability (sigma = 0.0) indicates that the data from the two studies are highly consistent.
