# Supplementary material for: Outcomes of Acute Kidney Injury in Melioidosis: A Systematic Review and Meta-Analysis
Source: Life (Basel). 2025 Jul 15;15(7):1108. doi: 10.3390/life15071108 (PMC12299289; doi:10.3390/life15071108)
Supplement: Supplementary file 1 [file life-15-01108-s001.zip › Table S1.pdf]

Supplementary Table S1: Search strategies of 3 databases

PubMed

Search number	Query	Search Details	Results
1	("Melioidosis"[Mesh] OR "Burkholderia pseudomallei"[Mesh] OR melioidosis OR "Burkholderia pseudomallei") AND ("Acute Kidney Injury"[Mesh] OR "acute renal failure" OR "AKI" OR "renal injury" OR "kidney injury") AND ("mortality" OR "outcome" OR "prognosis" OR "renal replacement therapy" OR "dialysis" OR "intensive care" OR "ICU" OR "hospital stay")	("Melioidosis"[MeSH Terms] OR "Burkholderia pseudomallei"[MeSH Terms] OR ("Melioidosis"[MeSH Terms] OR "Melioidosis"[All Fields]) OR "Burkholderia pseudomallei"[All Fields]) AND ("Acute Kidney Injury"[MeSH Terms] OR "acute renal failure"[All Fields] OR "AKI"[All Fields] OR "renal injury"[All Fields] OR "kidney injury"[All Fields]) AND ("mortality"[All Fields] OR "outcome"[All Fields] OR "prognosis"[All Fields] OR "renal replacement therapy"[All Fields] OR "dialysis"[All Fields] OR "intensive care"[All Fields] OR "ICU"[All Fields] OR "hospital stay"[All Fields])	10

SCOPUS

No.	Query	Results
1	(TITLE-ABS-KEY(melioidosis) OR TITLE-ABS-KEY("Burkholderia pseudomallei"))<br>AND<br>(TITLE-ABS-KEY("acute kidney injury") OR TITLE-ABS-KEY("acute renal failure") OR TITLE-ABS-KEY(AKI) OR TITLE-ABS-KEY("renal injury") OR TITLE-ABS-KEY("kidney injury"))<br>AND<br>(TITLE-ABS-KEY(mortality) OR TITLE-ABS-KEY(outcome) OR TITLE-ABS-KEY(prognosis) OR TITLE-ABS-KEY("renal replacement therapy") OR TITLE-ABS-KEY(dialysis) OR TITLE-ABS-KEY("intensive care") OR TITLE-ABS-KEY(ICU) OR TITLE-ABS-KEY("hospital stay"))	17

Embase

No.	Query	Results
#1	('melioidosis'/exp OR 'burkholderia pseudomallei'/exp OR melioidosis:ti,ab OR 'burkholderia pseudomallei':ti,ab) AND ('acute kidney injury'/exp OR 'acute renal failure':ti,ab OR 'aki':ti,ab OR 'renal injury':ti,ab OR 'kidney injury':ti,ab) AND ('mortality'/exp OR 'prognosis'/exp OR 'treatment outcome'/exp OR mortality:ti,ab OR outcome:ti,ab OR prognosis:ti,ab OR 'renal replacement therapy':ti,ab OR dialysis:ti,ab OR 'intensive care':ti,ab OR icu:ti,ab OR 'hospital stay':ti,ab)	44
