# Supplementary material for: Outcomes of Acute Kidney Injury in Melioidosis: A Systematic Review and Meta-Analysis
Source: Life (Basel). 2025 Jul 15;15(7):1108. doi: 10.3390/life15071108 (PMC12299289; doi:10.3390/life15071108)
Supplement: Supplementary file 1 [file life-15-01108-s001.zip › Table S2.pdf]

Supplementary Table S2. Eighteen studies were excluded from the final analysis.

[Ref]	First author [Ref]	Publication Year	Title	Reason for exclusion
[43]	Roa	2022	Hyponatremia in Melioidosis: Analysis of 10-year Data from a Hospital-Based Registry	duplicate population
[44]	Chua	2022	The Great Mimicker or the Great Masquerader?	lack of outcome data (non-associated)
[45]	Luvir	1998	Clinical features of renal diseases in South-East Asia	lack of outcome data (non-associated)
[46]	Mackintosh	2016	Goodpasture disease as a consequence of melioidosis	lack of outcome data (non-associated)
[47]	Martin	2025	Adverse reactions to trimethoprim/sulfamethoxazole for melioidosis eradication therapy: An evaluation of frequency and risk factors	lack of outcome data (non-associated)
[48]	Mukhopadhyay	2004	Bacteraemic melioidosis pneumonia: impact on outcome, clinical and radiological features	lack of outcome data (non-associated)
[49]	Nandurkar	2006	Melioidosis as a cause of multifocal osteomyelitis	lack of outcome data (non-associated)
[50]	Laklaeng	2024	Multi-locus sequence typing and genetic diversity of antibiotic-resistant genes and virulence-associated genes in *Burkholderia pseudomallei*: Insights from whole genome sequencing of animal and environmental isolates in Thailand	lack of outcome data (non-associated)
[51]	Patamatamkul	2017	A case-control study of community-acquired *Acinetobacter baumannii* pneumonia and melioidosis pneumonia in northeast Thailand: an emerging fatal disease with unique clinical features	lack of outcome data (non-associated)
[52]	Jabbar	2013	Melioidosis and the kidney	Review
[53]	Meumann	2024	Approach to melioidosis	Review
[54]	Norman	2024	The Evolving Global Epidemiology of Human Melioidosis: A Narrative Review	Review
[55]	Raja	2007	Melioidotic septic arthritis: A case report and literature review	Review & lack of outcome data (non-associated)
[56]	Keragala	2023	Efficacy and safety of co-trimoxazole in eradication phase of melioidosis; systematic review	Systematic review
[57]	Aravan	2022	Acute kidney injury and its outcome among melioidosis patients in a tertiary hospital of a north-eastern state of malaysia	Conference abstract
[58]	Grace	2022	Parathyroid hormone-independent hypercalcaemia secondary to granulomatous inflammation: could this be melioidosis?	Conference abstract
[59]	Janelle	2023	Atypical anti-gbm disease in association with systemic melioidosis	Conference abstract
60]	Tan	2022	*Burkholderia pseudomallei* (melioidosis) peritoneal dialysis peritonitis	Conference abstract
