# Supplementary material for: Outcomes of Acute Kidney Injury in Melioidosis: A Systematic Review and Meta-Analysis
Source: Life (Basel). 2025 Jul 15;15(7):1108. doi: 10.3390/life15071108 (PMC12299289; doi:10.3390/life15071108)
Supplement: Supplementary file 1 [file life-15-01108-s001.zip › Table S3.pdf]

Supplementary Table S3. Quality assessment of included studies using JBI checklists.

Study (Author, Year)	Study Type	JBI Score	Risk of Bias	Comments
Amali, 2024 [12]	Case Report	7/8 (88%)	Low	Lacks adverse event description
Arya, 2021 [13]	Case Report	8/8 (100%)	Low	Well-documented clinical progression
Alhatmi, 2020 [11]	Case Report	7/8 (88%)	Low	Limited detail on post-treatment course
Boyle, 2024 [14]	Case Report	6/8 (75%)	Moderate	Partial clinical history missing
Chang, 2020 [15]	Case Report	7/8 (88%)	Low	Clear renal presentation and outcome
Che Rahim, 2019 [17]	Case Report	8/8 (100%)	Low	Detailed diagnostics and recovery
Cossaboom, 2020 [18]	Case Report	8/8 (100%)	Low	Thorough diagnostic and outcome data
Fairhead, 2020 [19]	Case Report	6/8 (75%)	Moderate	Some missing diagnostic details
Gulati, 2022 [22]	Case Report	7/8 (88%)	Low	Fatal outcome well described
Gunasena, 2023 [23]	Case Report	7/8 (88%)	Low	Co-infection and dialysis documented
Hin, 2012 [25]	Case Report	6/8 (75%)	Moderate	Missing adverse event discussion
Jang, 2015 [27]	Case Report	8/8 (100%)	Low	Well-documented diagnosis and follow-up
Lim, 2022 [28]	Case Report	7/8 (88%)	Low	Sepsis and renal dysfunction outlined
Loh, 2017 [30]	Case Report	6/8 (75%)	Moderate	Limited intervention clarity
Meraj, 2019 [31]	Case Report	8/8 (100%)	Low	Persistent infection clearly described
Morelli, 2015 [32]	Case Report	7/8 (88%)	Low	Renal progression well explained
Stewart, 2021 [33]	Case Report	6/8 (75%)	Moderate	Limited follow-up and adverse data
Tamtami, 2017 [34]	Case Report	7/8 (88%)	Low	Imported case clearly documented
Wadwekar, 2018 [36]	Case Report	6/8 (75%)	Moderate	Limited treatment detail
Ganesan, 2019 [23]	Case Series	8/10 (80%)	Moderate	Limited on inclusion clarity

Study (Author, Year)	Study Type	JBI Score	Risk of Bias	Comments
Gouse, 2017 [21]	Case Series	7/10 (70%)	Moderate	Outcome definitions unclear
Gupta, 2021 [24]	Case Series	8/10 (80%)	Moderate	Some outcome variability
Jagtap, 2017 [26]	Case Series	7/10 (70%)	Moderate	Small sample, inconsistent detail
Liu, 2014 [29]	Case Series	8/10 (80%)	Moderate	Reported renal data clearly
Tran, 2022 [35]	Case Series	9/10 (90%)	Low	Strong case documentation
Warapitiya, 2021 [37]	Case Series	8/10 (80%)	Moderate	Limited outcome follow-up
Chanvitan, 2019 [16]	Case Series	8/10 (80%)	Moderate	Pediatric clarity, AKI rare
Prabhu, 2021 [5]	Cohort Study	10/11 (91%)	Low	Largest, well-designed study
Chou, 2007 [4]	Cohort Study	9/11 (82%)	Moderate	Older design, outcome robust
