# Supplementary figures and images for: Outcomes of Acute Kidney Injury in Melioidosis: A Systematic Review and Meta-Analysis
Source: Life (Basel). 2025 Jul 15;15(7):1108. doi: 10.3390/life15071108 (PMC12299289; doi:10.3390/life15071108)

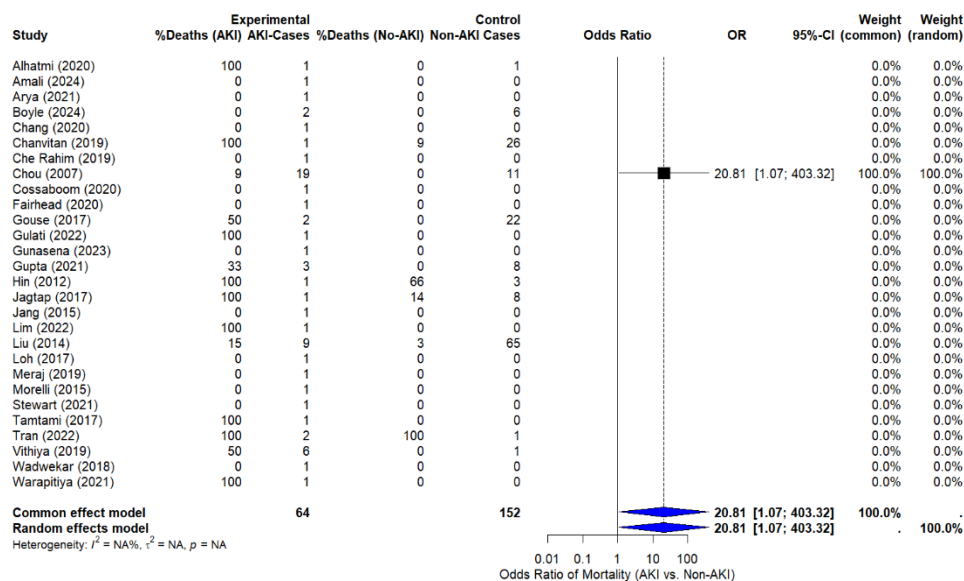

**Supplementary Figure S1.** Forest plot of the Sensitivity test by removing of Prabhu et al. (2021)

Supplement: Supplementary file 1 [file life-15-01108-s001.zip › Figure S1.pdf]

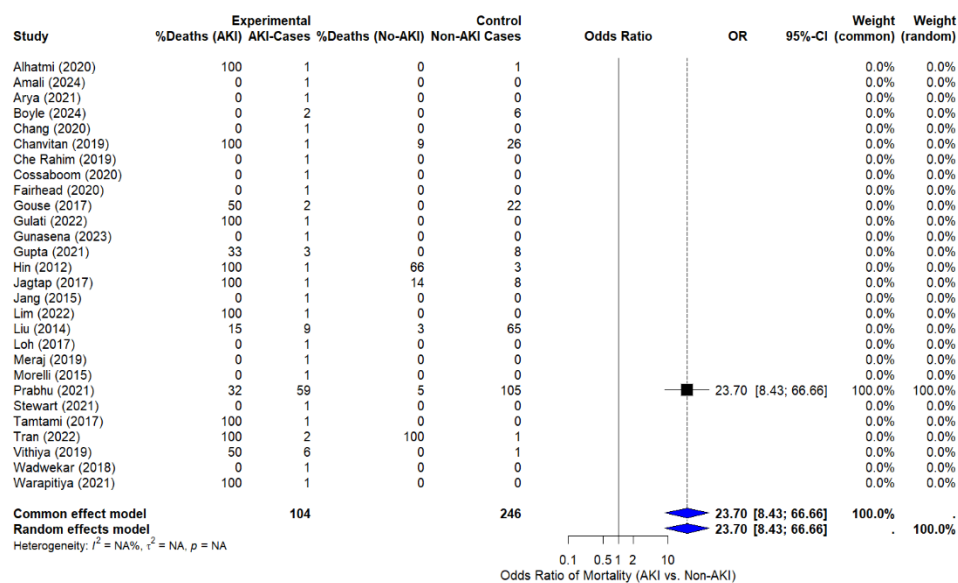

**Supplementary Figure S2.** Forest plot of the Sensitivity test by removing of Chou et al. (2007)

Supplement: Supplementary file 1 [file life-15-01108-s001.zip › Figure S2.pdf]
